# Supplementary material for: Understanding the origin of lithium dendrite branching in Li6.5La3Zr1.5Ta0.5O12 solid-state electrolyte via microscopy measurements
Source: Nat Commun. 2024 Sep 18;15:8207. doi: 10.1038/s41467-024-52412-4 (PMC11410937; doi:10.1038/s41467-024-52412-4)
Supplement: Supplementary file 1 — Supplementary Information [file 41467_2024_52412_MOESM1_ESM.pdf]

Supplementary Information

Can Yildirim[1], Florian Flatscher[2,3], Steffen Ganschow[4], Alice Lassnig[5], Christoph Gammer[5], Juraj Todt[5,6], Jozef Keckes[5,6], Daniel Rettenwander[2,3]

[1]European Synchrotron Radiation Facility, 71 Avenue des Martyrs, CS40220, 38043 Grenoble Cedex 9, France. [2]Department of Material Science and Engineering, NTNU Norwegian University of Science and Technology, Trondheim, Norway. [3]Christian Doppler Laboratory for Solid-State Batteries, NTNU Norwegian University of Science and Technology, Trondheim, Norway. [4]Leibniz-Institut für Kristallzüchtung, Berlin, Germany. [5]Austrian Academy of Sciences, Erich Schmid Institute of Materials Science, Leoben, Austria. [6]Chair of Materials Physics, Montanuniversität Leoben, Leoben, Austria.

Understanding the origin of lithium dendrite branching in $Li_{6.5}La_3Zr_{1.5}Ta_{0.5}O_{12}$ solid-state electrolyte via microscopy measurements

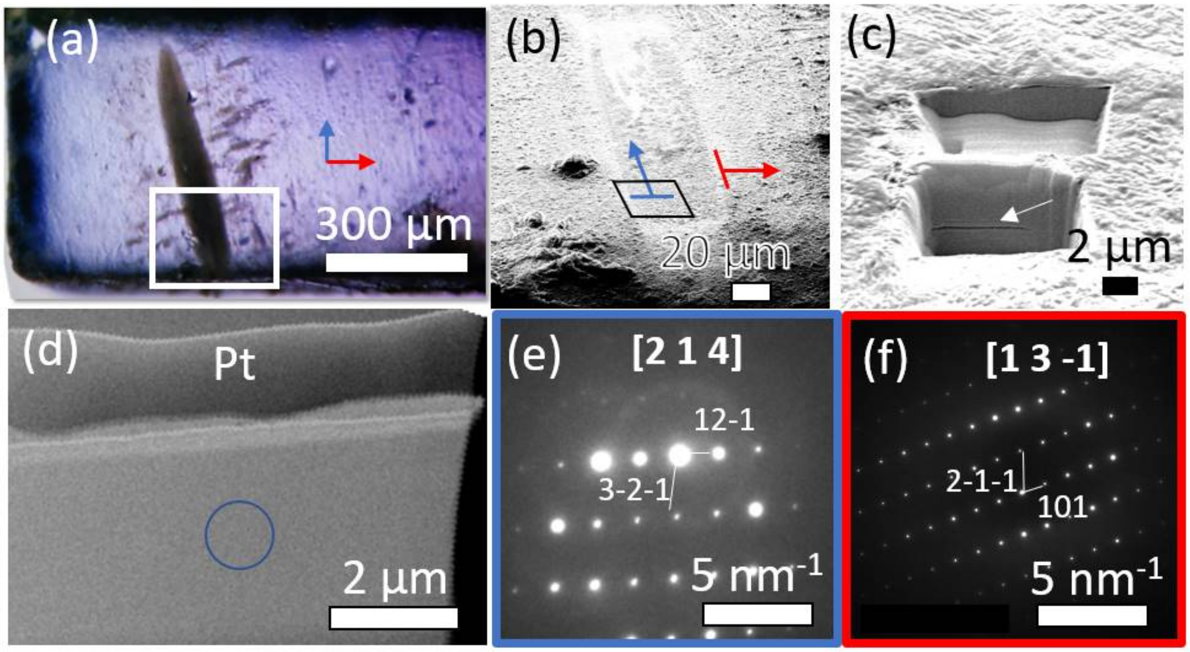

Supplementary Figure 1: TEM characterization of LLZO. (a) Light microscopy image showing the dendrite. (b) SEM image of the region indicated in (a). Within the dendrite (black square) a TEM lamella was prepared using FIB machining as shown by the SEM image in (c). A crack is visible at the position of the dendrite (indicated with an arrow). To analyze the microstructure in detail, BF-STEM imaging was carried out on the extracted lamella, showing that the sample is a near-perfect single crystal within the limitations of TEM technique. Only a thin surface oxide layer is visible below the protective Pt layer. To reveal the orientation, selected area diffraction was carried out (e). In addition, a TEM lamella was extracted normal to that, allowing to obtain the full crystallographic information (f). The resulting directions ([1 3 -1] and [2 1 4]) are indicated by blue and red arrows, respectively, in (a). The location of the TEM lamellas along with direction is given in (b).

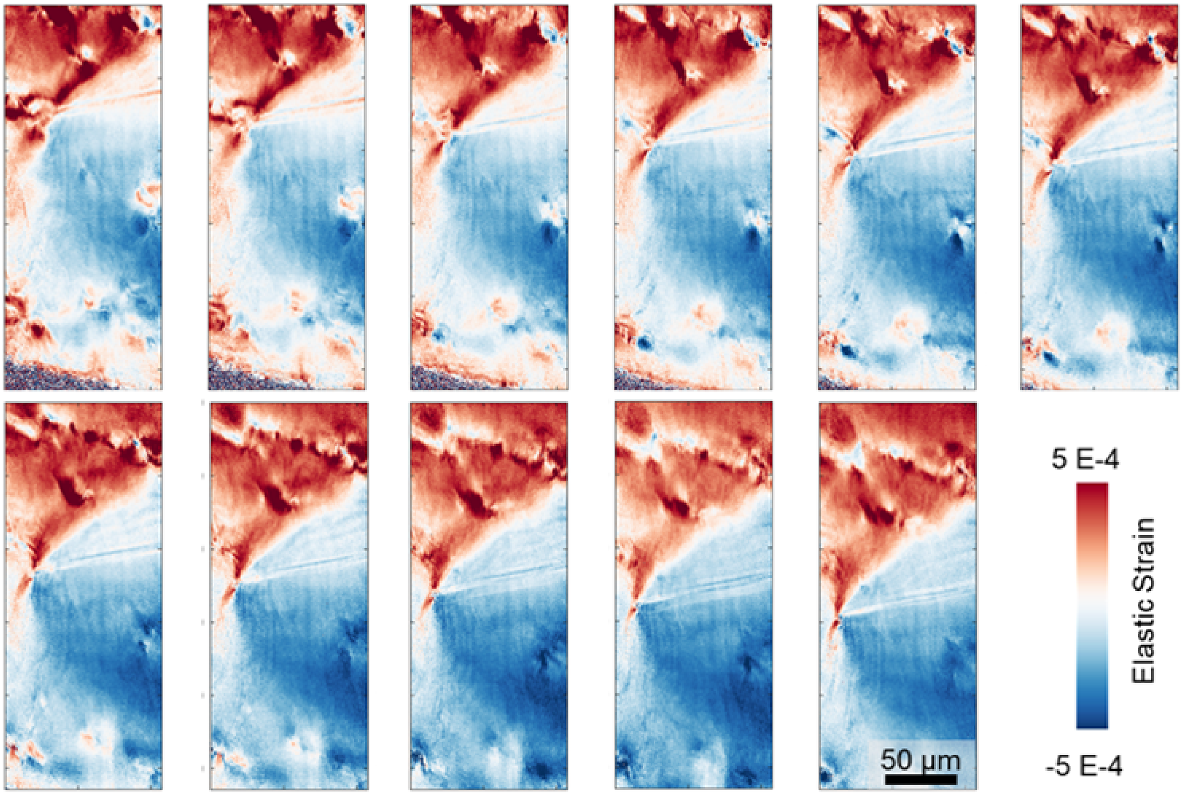

Supplementary Figure 2: Layered strain scans with 1 micron steps in the z direction, highlighting strain localization around the dendrite tip. Each image panel represents a 1 micron spacing from one another in the labs z coordinates, illustrating the strain heterogeneity within the bulk 3D structure.

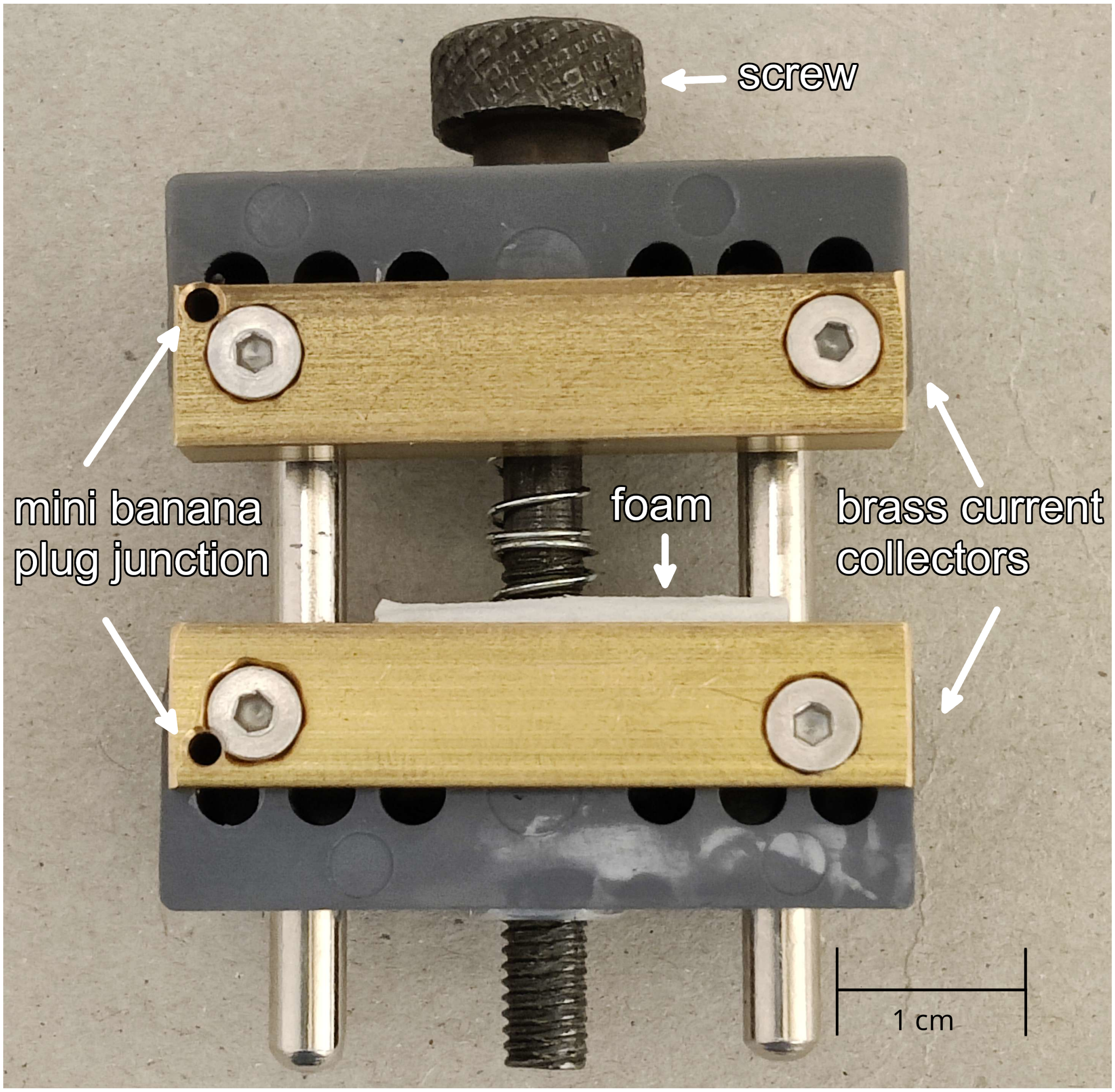

Supplementary Figure 3: Photographic picture of the lab-scale cell holder used for dendrites growth. The LLZTO sample, which was coated with LiSn alloy (30wt % Sn) on two opposing sides, was placed on the foam for better contrast in the microscope and fixed in place by closing the jaws with the screw. Once the brass jaw contacted the lithium on the sample it was tightened by one screw turn to achieve a good connection. It was determined that this applies a force of $3.3 \pm 0.5 * 10^{-3} N$. With a sample area of 3x0.2 mm the possible applied pressure on the sample is 5.5 ± 2.6 kPa. Current was delivered via the brass jaws.
