# Supplementary material for: Understanding the origin of lithium dendrite branching in Li6.5La3Zr1.5Ta0.5O12 solid-state electrolyte via microscopy measurements
Source: Nat Commun. 2024 Sep 18;15:8207. doi: 10.1038/s41467-024-52412-4 (PMC11410937; doi:10.1038/s41467-024-52412-4)
Supplement: Supplementary file 3 — Description of Additional Supplementary Files [file 41467_2024_52412_MOESM3_ESM.pdf]

Description of Additional Supplementary Files

Supplementary Movie 1: This video shows a Rocking Curve Measurement in Nearfield Diffraction Topography using Box-Beam Illumination. Brighter colors indicate higher intensity, revealing the dendrite's position within the box beam setup as the phi axis is rotated.

Supplementary Movie 2: This video provides a Detailed 3D Visualization of Dendrite-Dislocation Interactions in LLZTO. It displays a 40 µm progression along the z-axis, capturing the interactions between dendrites and dislocations within the ceramic matrix through DFXM orientation mapping.
